# Supplementary material for: Enhancing bioreactor arrays for automated measurements and reactive control with ReacSight
Source: Nat Commun. 2022 Jun 11;13:3363. doi: 10.1038/s41467-022-31033-9 (PMC9188569; doi:10.1038/s41467-022-31033-9)
Supplement: Supplementary file 1 — Supplementary Information [file 41467_2022_31033_MOESM1_ESM.pdf]

Enhancing bioreactor arrays
for automated measurements and reactive control with ReacSight
--Supplementary Information--

François Bertaux[1,2†#], Sebastián Sosa-Carrillo[2,1,3*], Viktoriia Gross[1,2,4], Achille Fraisse[2,1], Chetan Aditya[2,1,3], Mariela Furstenheim[1], Gregory Batt[2,1 †]*

1. Institut Pasteur, 28 rue du Docteur-Roux, 75015 Paris, France
2. Inria Paris, 2 rue Simone Iff, 75012 Paris, France
3. Université Paris Cité, 85 boulevard Saint-Germain, 75006 Paris, France
4. IAME Research Group, UMR 1137, Université Paris Cité and INSERM, 75018 Paris, France

* These authors contributed to this work equally.
† Correspondence: f.bertaux@lesaffre.com, gregory.batt@inria.fr
[#] Current address: Lesaffre International, 101 rue de Menin, Marcq-en-Baroeul, France

In Supplementary Note 1, we describe in more details the ReacSight strategy.

In Supplementary Note 2, we describe our bioreactor-based platforms enabling reactive optogenetic control and automated cytometry of yeast continuous cultures. Note that two such platforms exist, either with a custom bioreactor array or with Chi.Bio bioreactors.

In Supplementary Note 3, we provide additional information regarding yeast case studies: real-time control of gene expression with light, impact of nutrient scarcity on fitness and cellular stress, and control of a two-strain consortium composition.

In Supplementary Note 4, we provide a detailed description of the yeast strains and plasmids used in this study.

Code and files are available on the Git repository: https://gitlab.inria.fr/InBio/Public/reacsight.

Supplementary Note 1. The ReacSight strategy

ReacSight combines both software (section 1.1) and hardware (section 1.2) components.

1.1 Software

ReacSight is based on a versatile multi-instrument control architecture using python and the web-application framework Flask. First, Python is used to write instrument-specific APIs (Supplementary Figure 1) enabling programmatic control of each instrument (bioreactor components - pumps, optical density readings, LEDs – pipetting robot, measurement device, etc…). As illustrated with three examples, the Python ecosystem of open-source libraries allows to handle a variety of situations.

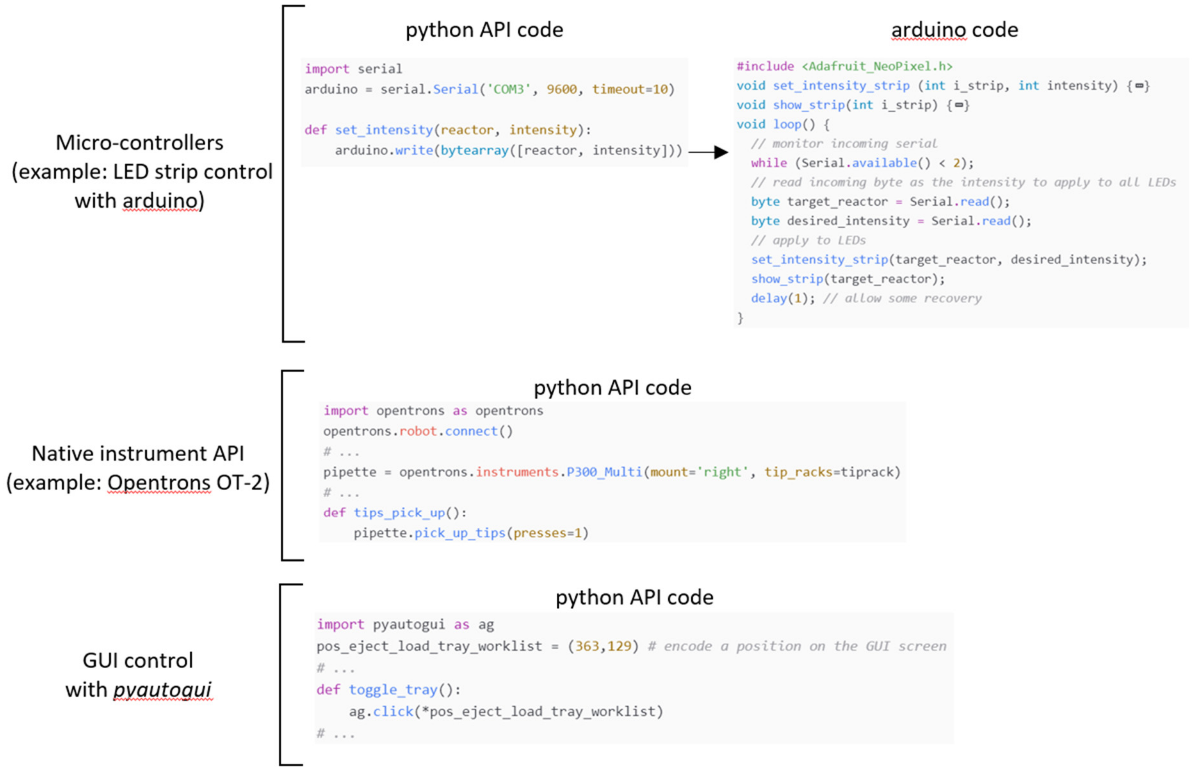

Supplementary Figure 1. Programmatic control of various instruments with Python. *Code snippets illustrating the versatility of python to control a diversity of instruments using open-source libraries such as pySerial (first example), pyautogui (last example) and opentrons for the Opentrons OT-2 pipetting robot (middle example).*

Next, the web-application framework Flask can be used to expose all instrument APIs to enable control from a single computer (Supplementary Figure 2). Writing the Flask app code for each instrument is straightforward and requires very little coding effort. For each instrument, the corresponding app should be running on a computer to which the main experiment control computer has access to. It could be another computer solely dedicated to the control of a given instrument (typically for the measurement device) connected to the local network or the same computer as the one running the experiment (for example for bioreactor components). For the Opentrons OT-2 robot, Flask can be installed and run from the Raspberry Pi computer inside the robot.

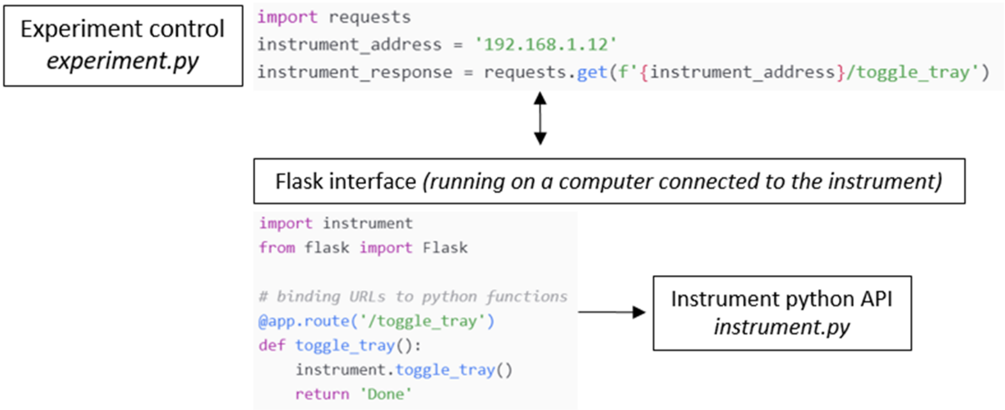

Supplementary Figure 2. Using Flask to expose instruments for simple access from a single experiment control computer. *Flask enables to develop very easily a web-app interface exposing the instrument control API to any computer who can access the computer running the Flask app (and controlling the instrument). At the level of experiment control, sending commands and receiving the corresponding data is easily done using the module requests. Note that in many situations, the same computer can be used for experiment control and instrument control.*

Finally, once multi-instrument control from a single computer with Python is achieved, the last step to start using a ReacSight platform is to write the code that execute desired experiments. At this stage, several platform-specific and experiment-specific challenges as well as choices to make could arise. Nevertheless, we propose a code architecture (i.e. a recipe for coding platform-specific experiments rather than the code itself) that should cover most scenarios and that should facilitate reactive experiment control and the re-use of code from one experiment to another. The main idea of this code architecture (Supplementary Figure 3) is to create a clear separation between 1) what is common across experiments and highly constrained by the platform design (main experiment loop) and 2) what can be made highly specific within an experiment and between different reactors in the same experiment (the reactor programs). With this architecture, it becomes natural to write the main experiment loop with a procedural style and the reactor programs with an object-oriented or functional style. To make this more concrete, we provide in the ReacSight Git repository an example implementation of an experiment loop and corresponding reactor programs. All experiments have been performed using the Windows 10 operating system.

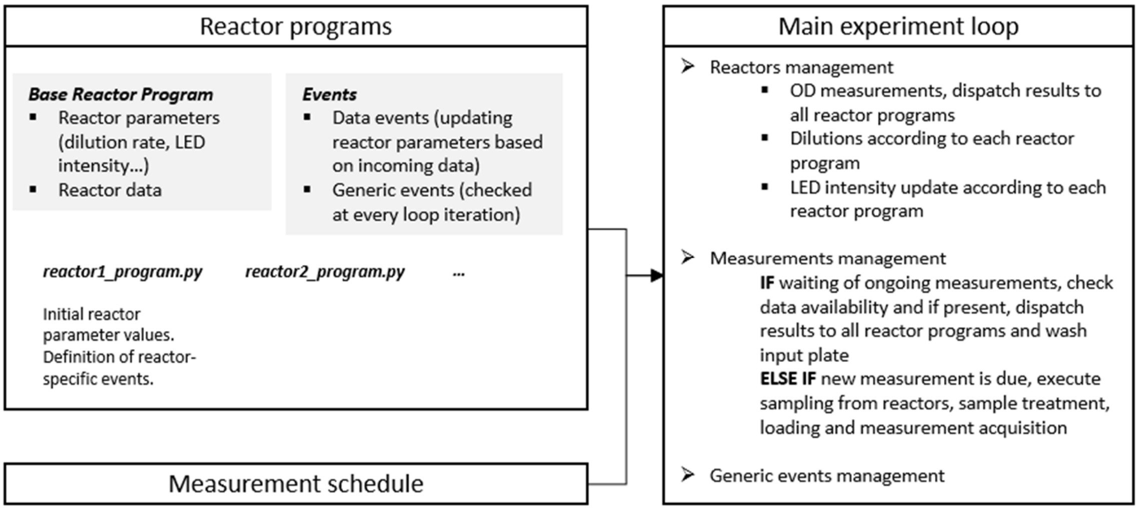

Supplementary Figure 3. A python code architecture for reactive experiment control of ReacSight platforms. *High-level description of the proposed architecture. The core of the experiment execution will be performed via a single and generic 'main experiment loop' (right). This is where the necessary synchronization for running parallel bioreactor experiments while measuring them using a single robot and measurement device will take place. On the other hand, all the experiment-specific*

and reactor-specific logic will be described through reactor programs (left). Each reactor program has given parameters than can be dynamically changed during the experiment through the definition of events.

1.2 Hardware

Core ReacSight hardware components are presented in Supplementary Figure 4. The two critical elements are the sampling and handling of bioreactor culture samples with the robot (Supplementary Figure 4A) and the positioning of the measurement device allowing access to the measurement input plate from the robot (Supplementary Figure 4B). Laser-cut pieces were obtained from 5mm thick acrylic plates cut with a TROTEC Speedy 300 machine ($C0_2$ laser). The convenience (Supplementary Figure 4C) waste funnel was 3D-printed with using an Ultimaker 3 machine using Ultimaker Material 2.85 PLA plastic thread. The 3D model was designed in Fusion 360.

This Opentrons OT-2 pipetting robot can be purchased for US$ 6800, including the multi-channel pipette.

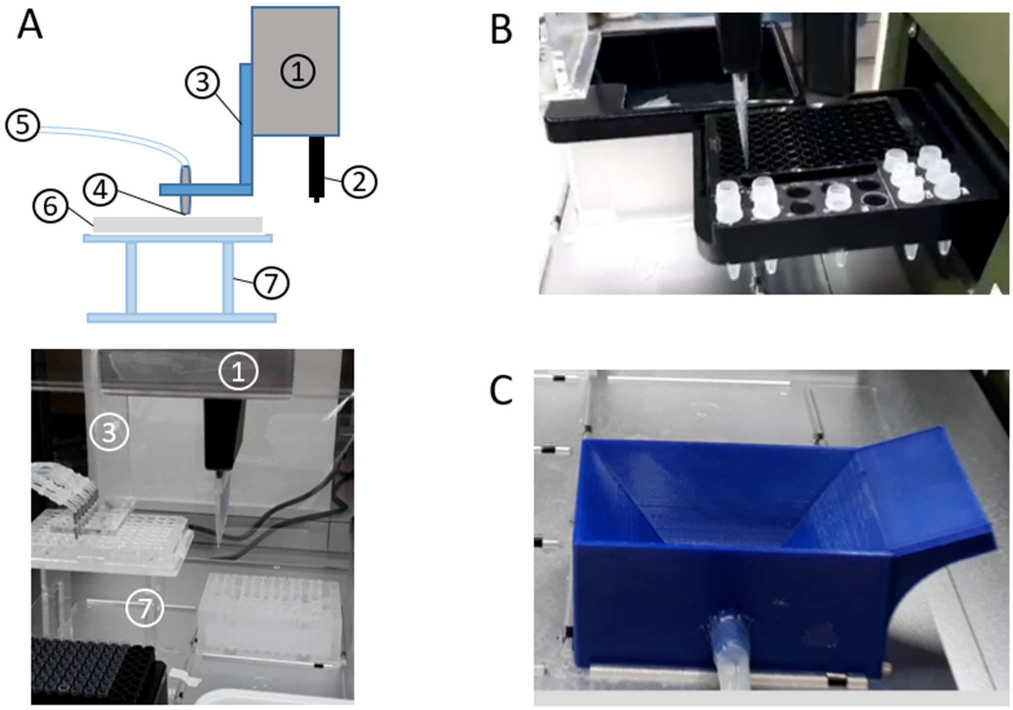

Supplementary Figure 4. Core ReacSight hardware components. (A) *Sampling and handling of bioreactor culture samples. Top: schematic showing 1 – X-Y moving arm of the robot, 2 – Z moving robot multi-pipette, 3 – sampling head (laser-cut acrylic), 4 – end of bioreactor sampling lines, 5 – sampling lines from bioreactors (should be pump-controlled), 6 – sampling plate, 7 – sampling plate holder (elevation required to prevent the sampling head to collide with other labware elements as the robot arm moves). Bottom: picture of the corresponding setup.* ***(B)*** *Robot access to the instrument input plate for treated samples loading and well washing. Typically, three columns of the input plate are accessible.* ***(C)*** *Convenience 3D-printed (PLA plastic) 'waste funnel' for disposal of significant liquid quantities.*

Supplementary Note 2. A ReacSight platform instance for reactive optogenetic control and automated cytometry for yeast continuous culture

We provide here details about our platform enabling reactive optogenetic control and automated cytometry for yeast continuous culture.

2.1 Optogenetic-enabled bioreactor setups

We present here the two optogenetic-enabled bioreactor setups used in the two versions of the platform.

2.1.1 A custom 16-reactor platform with optogenetic capabilities

We first extended a custom bioreactor platform designed and built for internal use at *Institut Pasteur* (E. Frachon, A. Jacquier and C. Saveanu). An overview of the platform is given in Supplementary Figure 5.

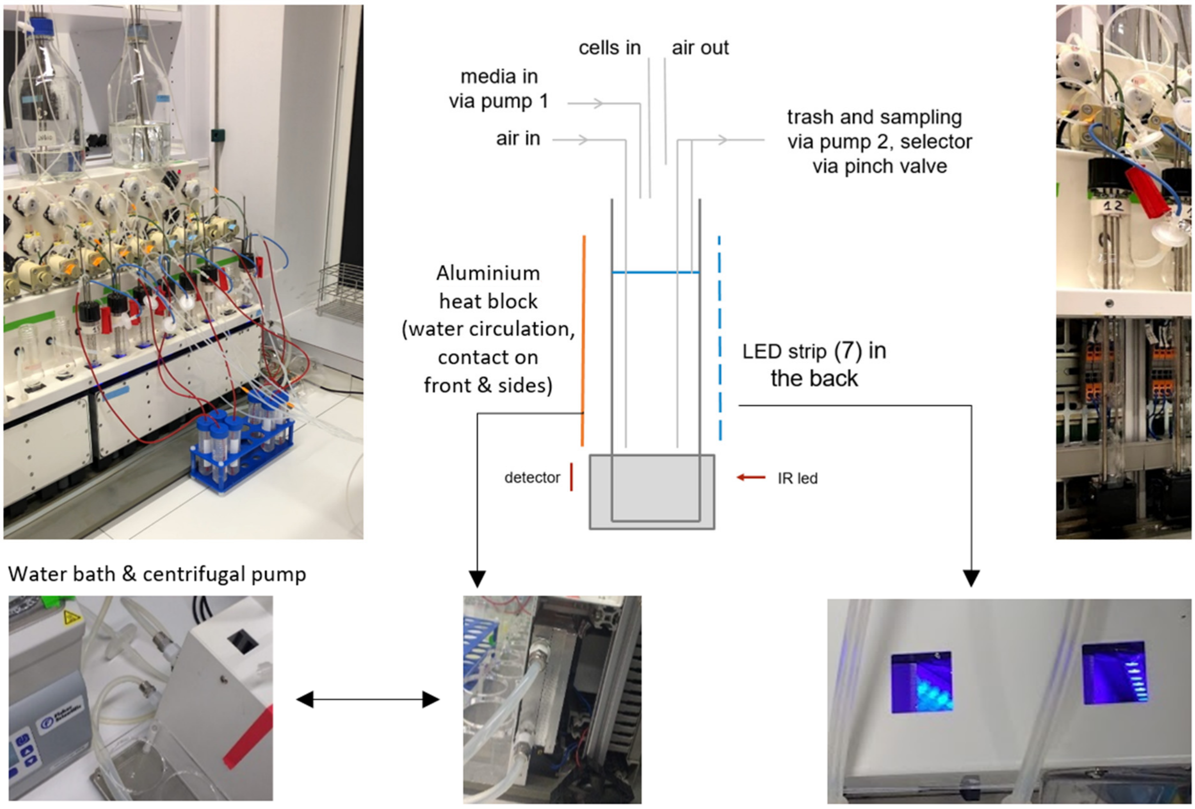

Supplementary Figure 5. Overview of the custom-16 reactor platform with optogenetic capabilities used in our lab. *Top-left: full view of the bioreactors, showing the vessels, media input bottles, pumps, etc. Top-right: scheme and picture of the vessel design in terms of air and liquid input/outputs. Mixing is achieved thanks to the bubbling caused by the air input. The scheme also highlights how optical density is measured, the position of a LED strip (bottom right picture) on one side of each vessel slot for optogenetic induction, and heating via contact to aluminium blocks on all other sides of the vessel (middle bottom picture). A closed water circulation system from (and back to) a water bath powered by a centrifuge pump maintains the temperature of the aluminium blocks.*

2.1.2 Setup with the Chi.Bio reactors

To demonstrate the modularity of the ReacSight strategy as well as illustrating that a platform enabling reactive optogenetic control and automated cytometry of microbial continuous culture can be easily assembled from low-cost, commercially available optogenetic-ready bioreactors, we constructed an

equivalent platform (Supplementary Figure 6A) based on the Chi.Bio system (Steele et al., PLoS Biology, 2020; purchased from LabMaker, Germany). The cost of the Chi.Bio system is US$ 5895 (including 8 reactors, 4 pumps per reactor and a controller board).

Because the native operating system of the Chi.Bio system is already a Flask app, very little changes to the original code (the modified version is available in the ReacSight Git repository) were necessary. We validated the functionality and performance of the using the light-induced gene expression strain, obtaining excellent reactor-to-reactor reproducibility of automated cytometry measurements upon a variety of light-induction profiles (Supplementary Figure 6B).

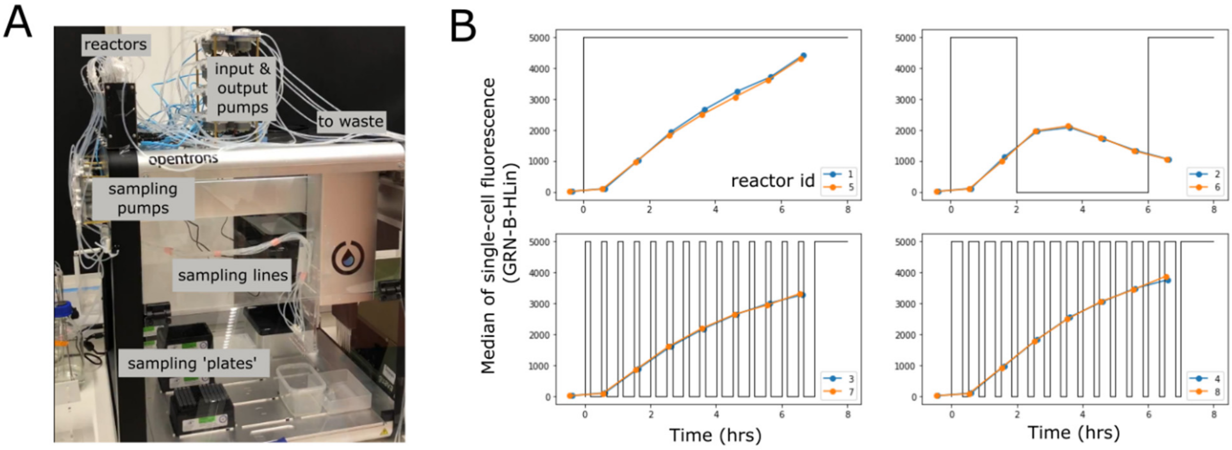

Supplementary Figure 6. Description and validation of the platform version using Chi.Bio reactors. *(A) Annotated picture of the setup. A few technical differences exist between the two platforms. For example, differences in pumps flow rate resulted in a stronger output stream compared to the other version, requiring us to adapt the type of sampling plates (from regular 300 µL 96-well plate to deeper, larger-volume plates) to eliminate undesired 'splashing'. Also, because with the Chi.Bio setup, three pumps per reactor can be used, a completely separate output line is used for excess volume removal and goes directly to a waste bin. The dead volume is similar for both platforms (around 2 mL). (B) Validation experiments. The eight reactors were run in parallel with the light-induced gene expression strain (same as in Figure 2D and Figure 3 of the main text). Four different ON/OFF blue light induction patterns (dark lines) were applied in pairs of reactors to assess functionality of light-induced gene expression as well as reactor-to-reactor reproducibility. Cytometry data acquisition was performed automatically every hour.*

2.2 Metrology and automated analysis of cytometry data from yeast cultures

We verified that with fixed cytometry acquisition settings as well as fixed turbidostat culture conditions, raw fluorescence distributions of single-color strains are reproducible from week-to-week (Supplementary Figure 7A). After autofluorescence substraction, each single-color strain gives us a spectral signature across all fluorescence channels of our instrument, and these signatures are also highly reproducible week-to-week and even month-to-month (Supplementary Figure 7B).

Our automated analysis pipeline to go from raw fluorescence data to size-normalized fluorophore levels (i.e. concentrations for cytosolic fluorophores) is presented in Supplementary Figure 7B (gating based on forward and side scatter data, doublet removal based on deviation from linearity between the *Area* and *Height* of the forward scatter) and Supplementary Figure 7C and D (per-cell fluorescence deconvolution). After gating and deconvolution, fluorophore levels are divided by the forward scatter to yield size-normalized fluorophore levels. Finally, to express these values in Relative Promoter Units, they are normalized by equivalent numbers obtained once and for all with single-color strains with pTDH3-driven constructs.

The choice of the forward scatter value to size-normalize fluorophore levels has been motivated as follows. FSC-based size normalized was used in previous works performing real-time control of gene

expression using model-based approaches in yeast (Milias-Argeitis et al, 2011) and bacteria (Milias-Argeitis et al., 2016). We note however that other works (Zuleta et al., 2014; Harrigan et al., 2018; Swaffer et al., 2021) used SSC to size-normalize their cytometry data. Therefore, consistently with Tzur et al. (2011), there is no perfect proxy for yeast cell volume in optical cytometry-based measurements, and what are good proxies might depend on the instrument and conditions. We therefore looked at our own data to decide how to reduce as much as possible the confounding effect of cell volume variability.

In our data, we found that normalizing by FSC reduced the cell-to-cell variability of constitutively expressed fluorophore slightly more than normalizing by SSC (coefficient of variation changing from 0.4 to 0.27 vs 0.4 to 0.29 for pTDH3-driven mScarlet-I expression). This suggests that FSC correlates with cell volume slightly better than SSC (with our instrument and growth conditions), as cell size is expected to be a major contributor to cell-to-cell variability in protein levels for a constitutively expressed gene. We also inspected the steady-state distributions of both the FSC and SSC of turbidostat cultures and found that the FSC distributions generally resembled more published volume distributions measured with a Coulter counter (Jorgensen et al., 2002) than SSC distributions. We therefore used FSC throughout our study.

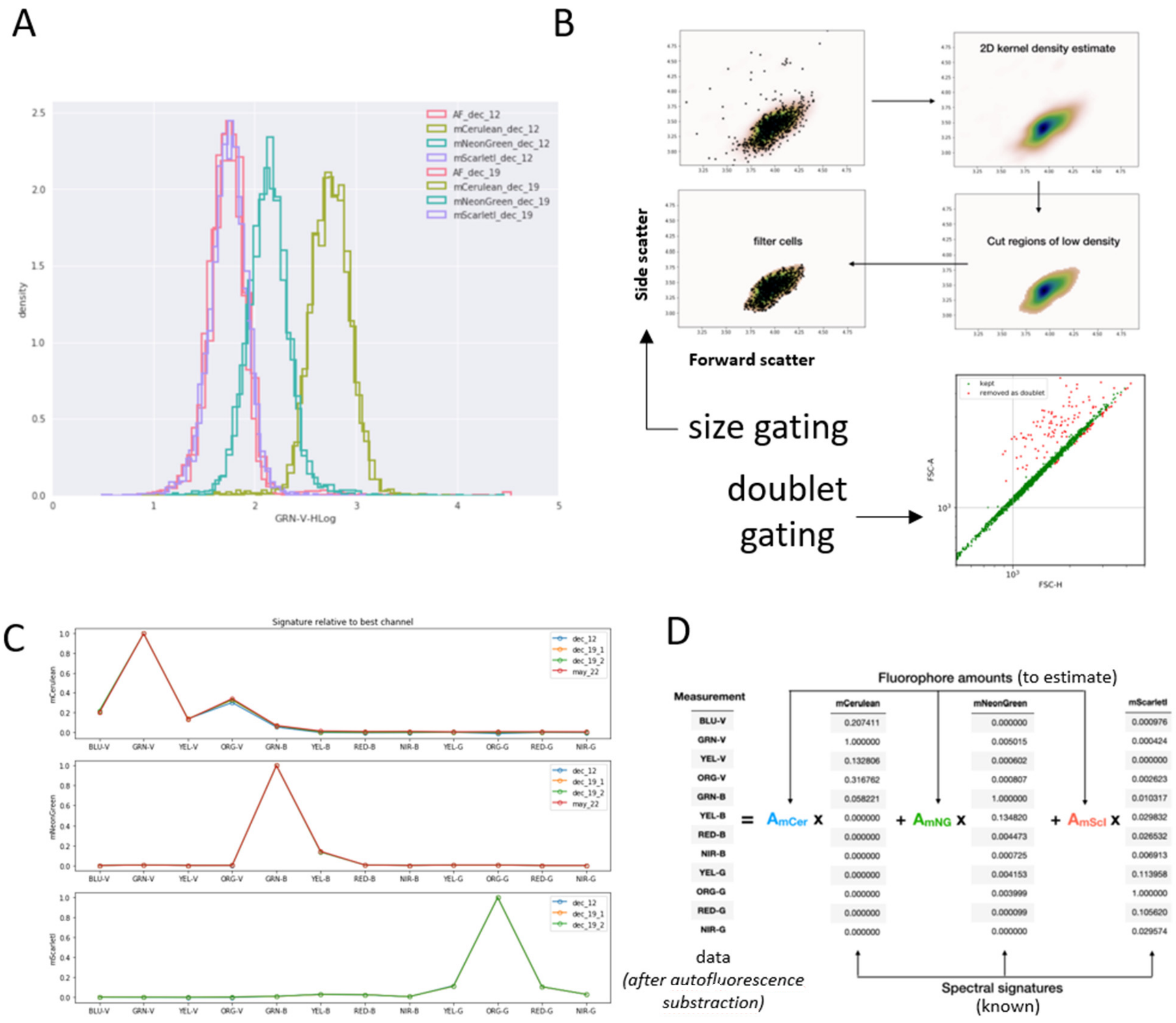

Supplementary Figure 7. Metrology and automated analysis of cytometry data from yeast cultures. *(A) The same turbidostat culture experiments for single-color strains were performed one week apart and cultures were measured with our*

cytometer with fixed acquisition settings. Here raw distributions for one channel is shown. Distributions precisely overlapped week-to-week. (B) Description on the size gating procedure, based on local density in the FSC – SSC space, and the doublet removal procedure, based on the comparison between FSC Area and Height. In total, our gating procedure has therefore two parameters: the size gating threshold (between 0 and 1, cut-off of the local density relative to the maximum density) and the doublet removal threshold (deviation from linearity between FSC Area and Height normalized to the dataset deviation). (C) Spectral signatures for the three fluorophores mCerulean, mNeonGreen and mScarlet-I (i.e., relative channel intensity after autofluorescence substraction for a corresponding single-color strain) are also highly reproducible week-to-week and even month-to-month. (D) Per-cell deconvolution is performed by solving the depicted linear system with Numpy linear algebra least-squares solver. Note that the residual error can be used to inform on the quality of the deconvolution on a per-cell basis.

2.3 Using the OT-2 robot for automated cytometry

The setup enabling automated cytometry with the OT-2 robot is described in more details in Supplementary Figure 8. We used fluorescent beads to validate the efficiency of the robot-based washing of the input plate wells inside the cytometer. Based on conservative estimates of the dilution efficiency of a single wash step, we decided to perform 3 consecutive washes after every cytometry acquisition, because less than one cell out of 600-800 is predicted to be left in the acquisition plate. Because this small number of remaining cells is diluted in pure water, they won't be able to grow and hence will have a negligible impact on future timepoints. This is verified in practice by gene expression induction experiments, where almost all cells acquired several hours after induction have high expression levels, despite the potential presence of pre-induction carry-over cells.

We also tested a setup where the same 8 tips are used for all timepoints of an experiment, rather than new tips being picked-up at every timepoint. We did not notice any significant difference in the data acquired that way, and it strongly reduces the use of tips, running cost and environmental impact of experiments.

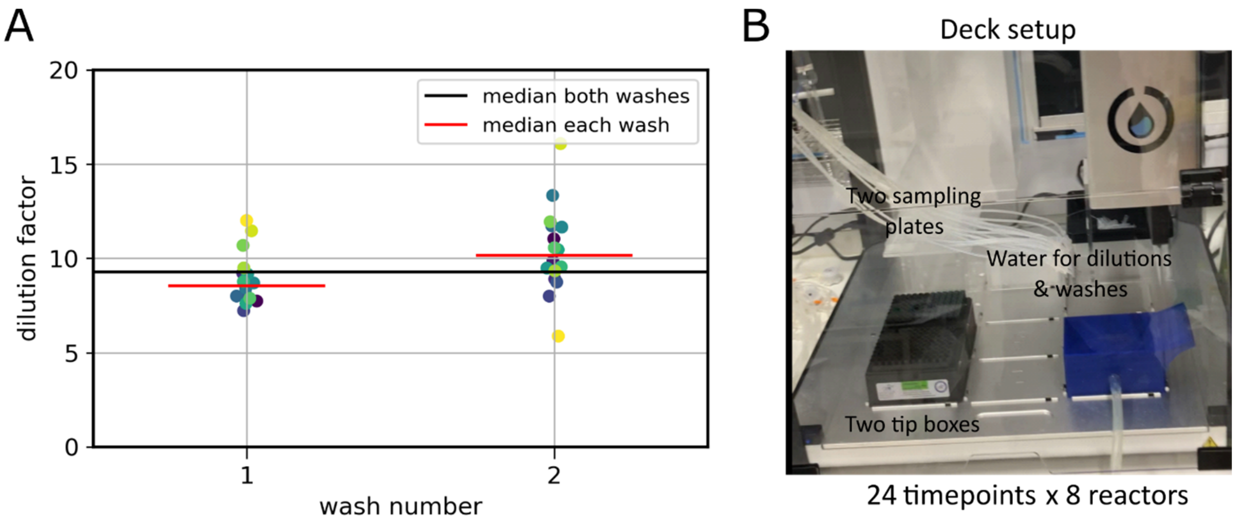

Supplementary Figure 8. Cytometer plate washing and deck setup for automated cytometry with the OT-2 robot. *(A) Validation of the efficiency of washing steps using fluorescent beads. Two plate columns (16 wells) containing a solution with fluorescent calibration beads were analyzed with the cytometer before and after each of two consecutive washes with the OT-2 robot, in order to estimate the dilution efficiency of each wash via the observed decrease in beads events per volume. The observed beads dilution factor is shown for the two washes; one dot corresponds to one of the 16 wells. The typical dilution factor is around 9, leading to total dilution factor of around 700 with three consecutive washes (as performed for all experiments of this paper). Higher efficiency can be obtained by further minimizing dead volume. (B) Our typical deck setup for starting an experiment. With two sampling plates and two tip boxes, we can perform 24 timepoints for each of 8 reactors in parallel without any human intervention. Renewing tips, plates and water reservoir takes only a few minutes and allow to continue an experiment over several days.*

In Supplementary Figure 9, we show that upon full light induction, the light-induced gene expression system we constructed can lead to expression levels slightly above the one observed with the strong

constitutive promoter pTDH3. This slightly higher expression level was consistently observed in many other experiments.

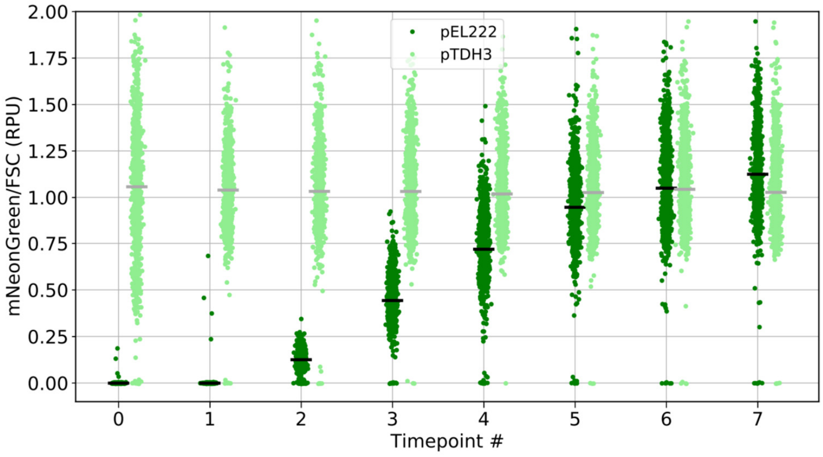

***Supplementary Figure 9. Full light induction eventually leads to expression levels slightly above the levels obtained with pTDH3**. The two reactor timecourses shown here were part of the same parallel experiment and are the same as shown in main text Figure 2c (pTDH3-mNeonGreen) and 2d (reactor with the light pattern depicted in dark blue, which is full ON for the first 8 timepoints). Strains are yIB88 (pTDH3-driven mNeonGreen expression) and yIB86 (light-driven gene expression).*

Supplementary Note 3. Additional information on ReacSight case studies

In this section we present additional information regarding the three ReacSight case studies described in this paper.

3.1 Real-time control of gene expression using light: induction strength, reproducibility and robustness

In Supplementary Figure 10, we show, by repeating experiments of main text Figure 3c several months later with two targets in quadruplicates, that real-time control of gene expression with light using a model predictive control algorithm on our ReacSight platform instance is highly robust and reproducible.

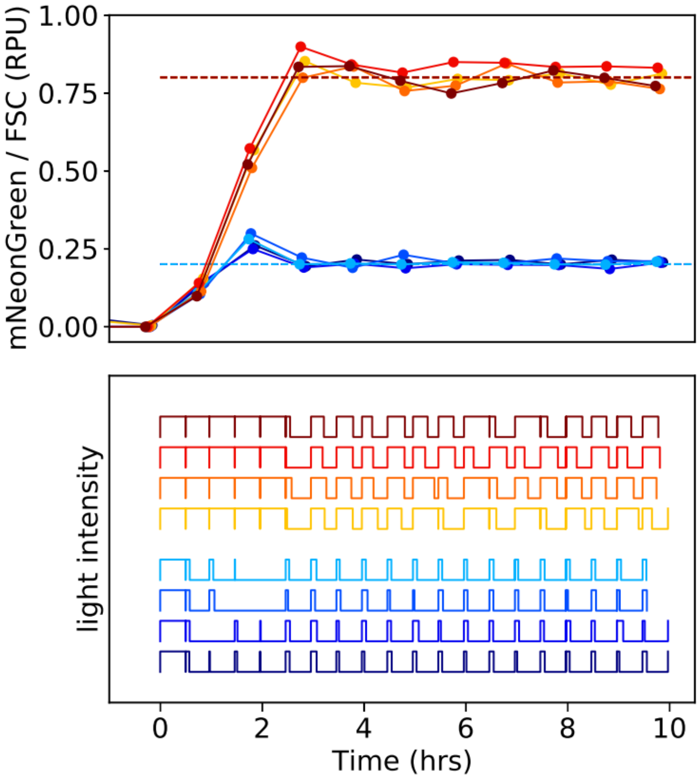

Supplementary Figure 10. Reproducibility and robustness. *An additional real-time control experiment as presented in Figure 3c of the main text was conducted, using this time the full 8-reactor capacity to perform 4-replicates of control experiments with the two extreme gene expression targets of Figure 3c, in a single experiment run.*

In Supplementary Figures 11 and 12, we represent the internal levels of a protein, mNeonGreen, in a secreted or non-secreted form, together with the corresponding levels of unfolded protein stress in cells, as reported by a unfolded protein stress reporter promoter (pUPR) controlling the expression of mScarlet-I. These data complement the data shown on Fig 3d of the main text.

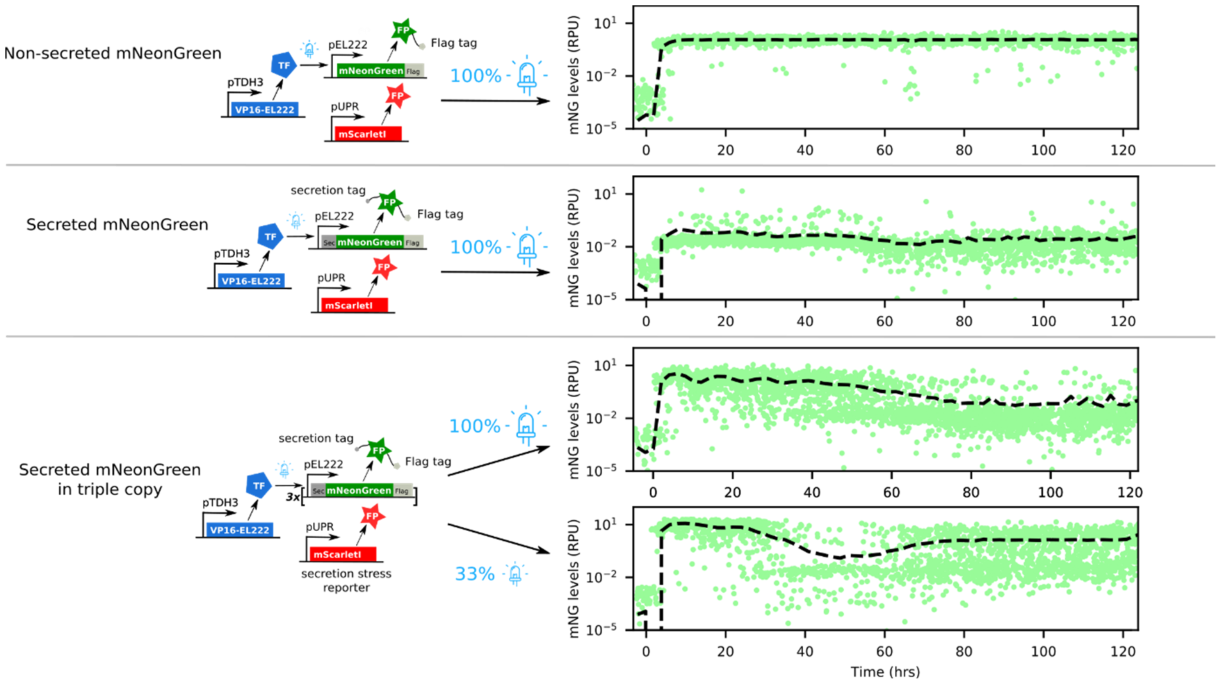

Supplementary Figure 11. Long-term stability of the expression system and impact of protein secretion: internal protein levels. Cells expressing an EL222-driven mNeonGreen fluorescent reporter, secreted or not, and present in one or three copies, are grown in turbidostats for 5 days with cytometry measurements every 2 hours. Cells also harbor a fluorescent reporter for secretion stress, mScarlet-I. Data show single-cell mNeonGreen levels as a function of time. The mean is represented by a black dotted line. To ease readability, only 500 events are represented per time point (5000 were acquired).

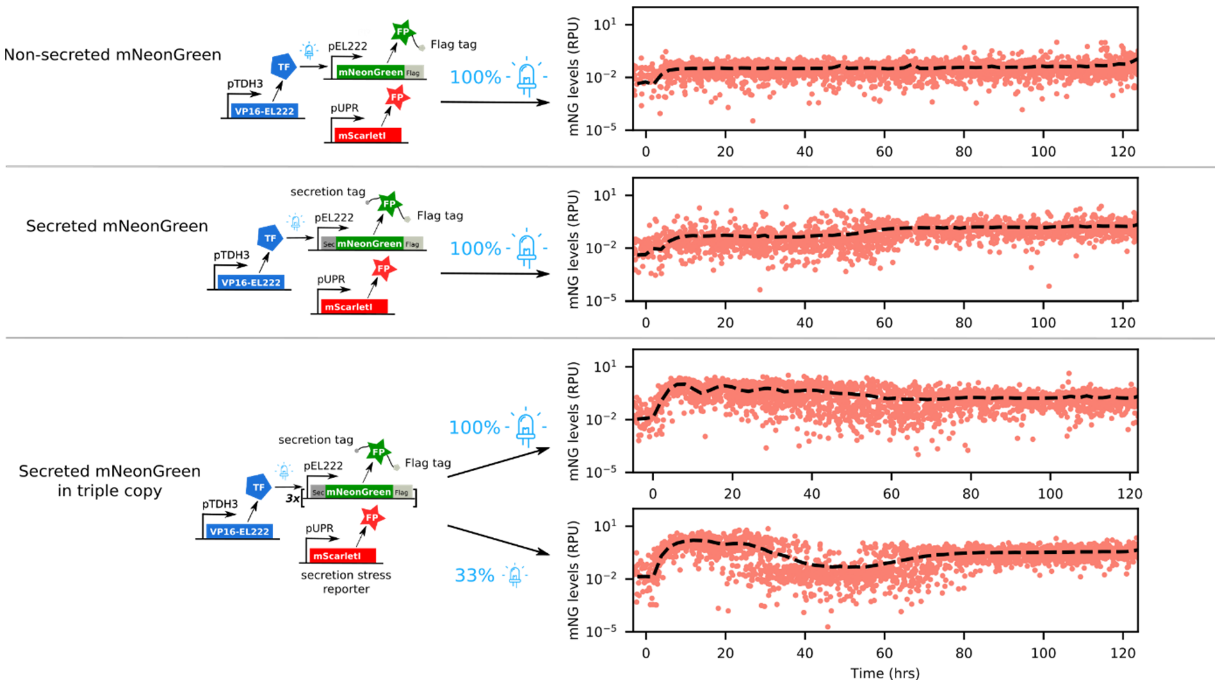

Supplementary Figure 12. Long-term stability of the expression system and impact of protein secretion: stress reporter levels. Cells expressing an EL222-driven mNeonGreen fluorescent reporter, secreted or not, and present in one or three copies, are grown in turbidostats for 5 days with cytometry measurements every 2 hours. Cells harbor a fluorescent reporter

for secretion stress, mScarlet-I. Data show single cell mScarlet-I levels as a function of time. The mean is represented by a black dotted line. To ease readability, only 500 events are represented per time point (5000 were acquired).

3.2 Impact of nutrient scarcity on fitness and cellular stress: mono-culture controls to assess the reliability of strain identification

In this case study, we used competition experiments of turbidostat cultures with automated cytometry to investigate the impact of nutrient scarcity on fitness and the UPR stress response. The two strain genotypes (Supplementary Figure 13, A and B) should allow in principle to identify the genotype of single-cell via mCerulean expression levels, enabling both the analysis of growth rate differences between the two strains via the evolution of their ratio and the extraction of strain-specific UPR stress response via the mScarlet-I expression levels.

We ran single-strain control experiments to validate the performance of mCerulean-based single-cell genotyping (Supplementary Figure 13, C and D). The results show that mCerulean expression in the WT strain is observed in almost all cells and is stable over 2 days, while almost no cells in mutant-only cultures are identified as mCerulean expressing cells. We analyzed the range of ratios between the two strains that are resolvable given the small occurrence of misattribution events and found it to be at least [0.01 – 100] (Supplementary Figure 13E).

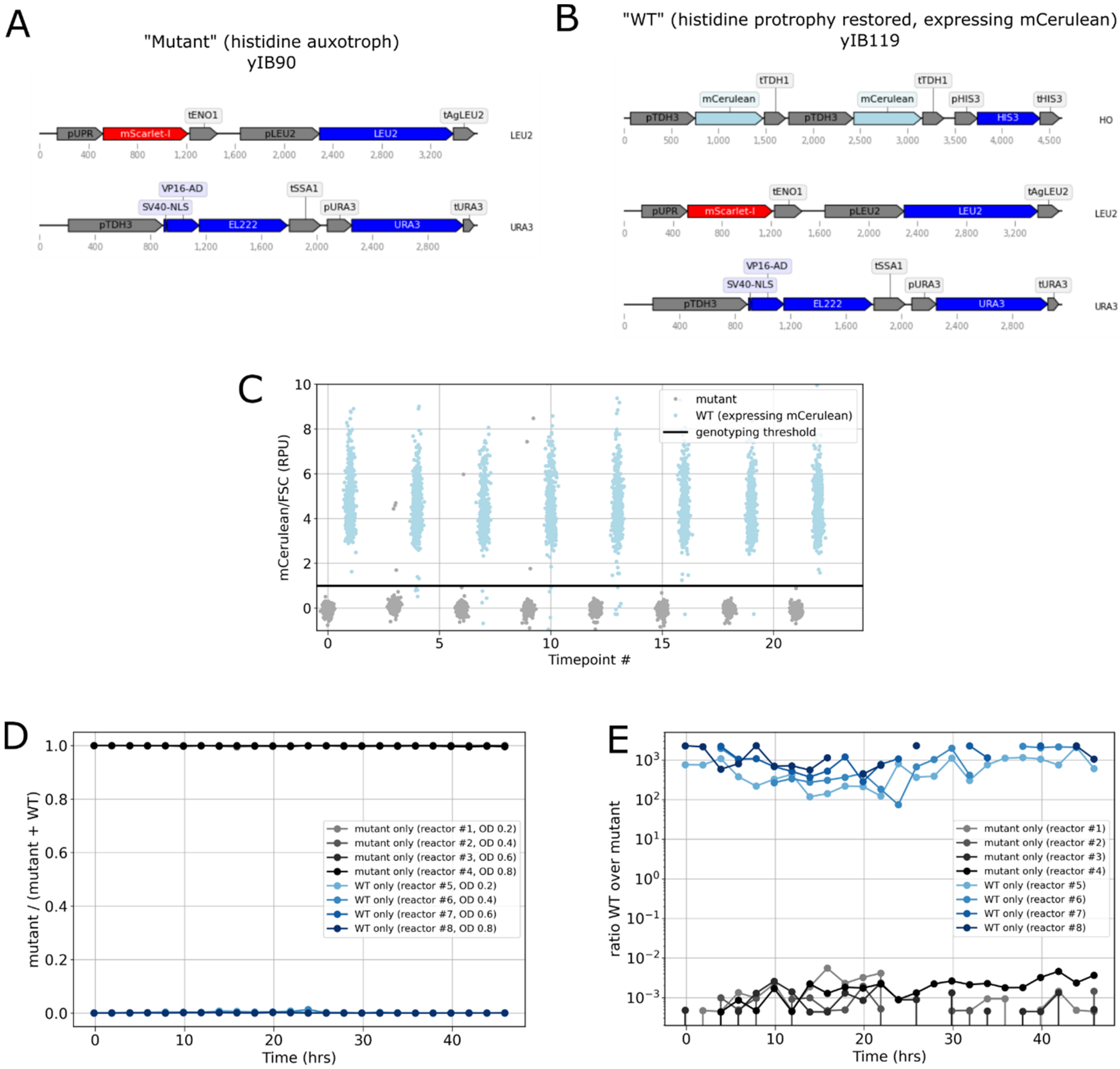

Supplementary Figure 13. Validation of the strain identification strategy. (A,B) *Illustration of the genotypes of the "mutant" strain (A) and the "WT" strain (B). Shown are integration sequences on BY4741 background. The "WT" strain was obtained from the "mutant" strain by transforming a cassette restoring histidine prototrophy and leading to constitutive expression of mCerulean.* ***(C,D,E)*** *Eight mono-culture control experiments (4 culture per strain) were run in parallel in turbidostat mode (4 different ODs per strain) to evaluate the performance and stability of the strain identification strategy.* ***(C)*** *Illustration of cytometry-based identification of the two strain genotypes, via the mCerulean/FSC metric after gating and deconvolution. Representative timepoints are shown.* ***(D,E)*** *Validation of the strain identification strategy on all cytometry timepoints for the 8 control cultures.* ***(D)*** *Detected fraction of mutant cells as a function of time for mutant only cultures (grey to black) and WT only cultures (shades of blue). Fractions are closed to the desired values of 1 and 0.* ***(E)*** *Same data with the ratio WT over mutant plotted instead (log-scale). This shows that ratio estimates are trustworthy in the range 0.01 – 100, while outside this range rare misattribution events can prevent accurate estimation of the true ratio.*

3.3 Real-time control of a two-strain consortium using nutrient scarcity: investigating limitations in controllability

Here we describe our investigation of the factors limiting the performance of the original two-strain consortium control experiment presented in Figure 4c of the main text. After identifying a potential role of the medium composition on the long-term stability of the "slow" strain slow growth phenotype, we decided to use a better suited medium composition. With this new medium, we studied the growth

rate of the two strains in mono-cultures for different conditions of light and OD (Supplementary Figures 14 and 15), we re-characterized their competition dynamics as a function of OD (Supplementary Figure 16), and we performed another control experiment (Supplementary Figure 17).

The rationale for this case study is to compete a histidine auxotroph strain (yIB88) with a slow but histidine prototroph strain (yIB135, slow phenotype triggered by light because of induction of burdening protein secretion). Detailed genotypes of these two strains in shown in Supplementary Figure 14A and C. This should allow bi-directional steering based on setting the OD setpoint of the turbidostat-operated co-culture when using low histidine medium: indeed, at low OD, the growth rate of the auxotroph strain should be close to wild-type and thus higher than the slow prototroph strain. Conversely, at high OD, histidine scarcity will strongly reduce the auxotroph strain growth rate, allowing the slow prototroph strain to outgrow it.

However, as can be seen in Figure 4c of the main text, bi-directional steering does not seem to be maintained over the whole course of the experiment: in several reactors, the strain ratio is near constant despite the OD being kept at the lowest value of the allowed range (0.5), which was supposed to favor the auxotroph strain. We reasoned that this could be caused by the recovery of a normal growth rate by the "slow" prototroph strain (burden adaptation). This was surprising because no such recovery was observed in our original characterization experiments of the slow prototroph strain. However, these original experiments were done with our standard medium in the lab: a synthetic complete medium based on a Formedium yeast nitrogen base (Formedium CYN6510) and supplement mixture (Formedium DCS0019). In contrast, for reason of reagent availability in the lab and purchasing issues, we used a Sigma yeast nitrogen base (Y0626) and supplement mixture (Y1751) to make low histidine medium for the two-strain control experiment. Because the Sigma supplement mixture contains more amino acids (in quantity and diversity) than the Formedium supplement mixture, we suspected it could facilitate the adaptation to expression burden.

We therefore decided to re-start a thorough characterization of these two strains and their competition dynamics in a new low histidine medium based on Formedium yeast nitrogen base (Formedium CYN6510), Formedium supplement mixture without histidine (Formedium DCS0079), and with added histidine (Sigma 53319).

Characterization of growth phenotypes in mono-cultures in new low histidine media

We used mono-cultures of the two strains at different conditions of light induction and OD setpoints to validate that the desired phenotypes are achieved. The effective dilution rate needed to maintain cultures at fixed OD are used to inform about growth rate, as at steady state the dilution rate per culture volume should equal the growth rate.

The auxotroph strain growth rate is not affected by a change of OD setpoint from 0.2 to 0.5 but it is reduced approximately 3-fold when the OD setpoint is changed from 0.2 to 0.8 (Supplementary Figure 14A and B). This phenotype is desired as it means that OD setpoint can be used to modulate the auxotroph strain growth rate. The cytometry data show that the cell population is homogenous in morphology (FSC-SSC) and fluorescence (GRN-B – ORG-G) at OD setpoints of 0.2 and 0.5 (Supplementary Figure 14C, left and middle), with the expected high GRN-B fluorescence from the mNeonGreen constitutive expression and low ORG-G fluorescence because of the absence of other fluorophores. Under higher histidine scarcity (OD setpoint of 0.8, Supplementary Figure 14C, right), a distinct population of presumably non-viable/dying cells appears, exhibiting both lower FSC and loss of GRN-B fluorescence.

As desired, the slow strain growth rate is reduced when light is turned ON (Figure 14E), by approximately 25-30 %, and this reduced growth rate is not affected when OD is changed from 0.2 to 0.5 or to 0.8. Importantly, the reduced growth rate is stable over at least 40 hours. Cytometry data clearly show an homogenous induction of the UPR response when light is turned ON, as nearly all cells display a high level of ORG-G fluorescence under light (Figure 14F).

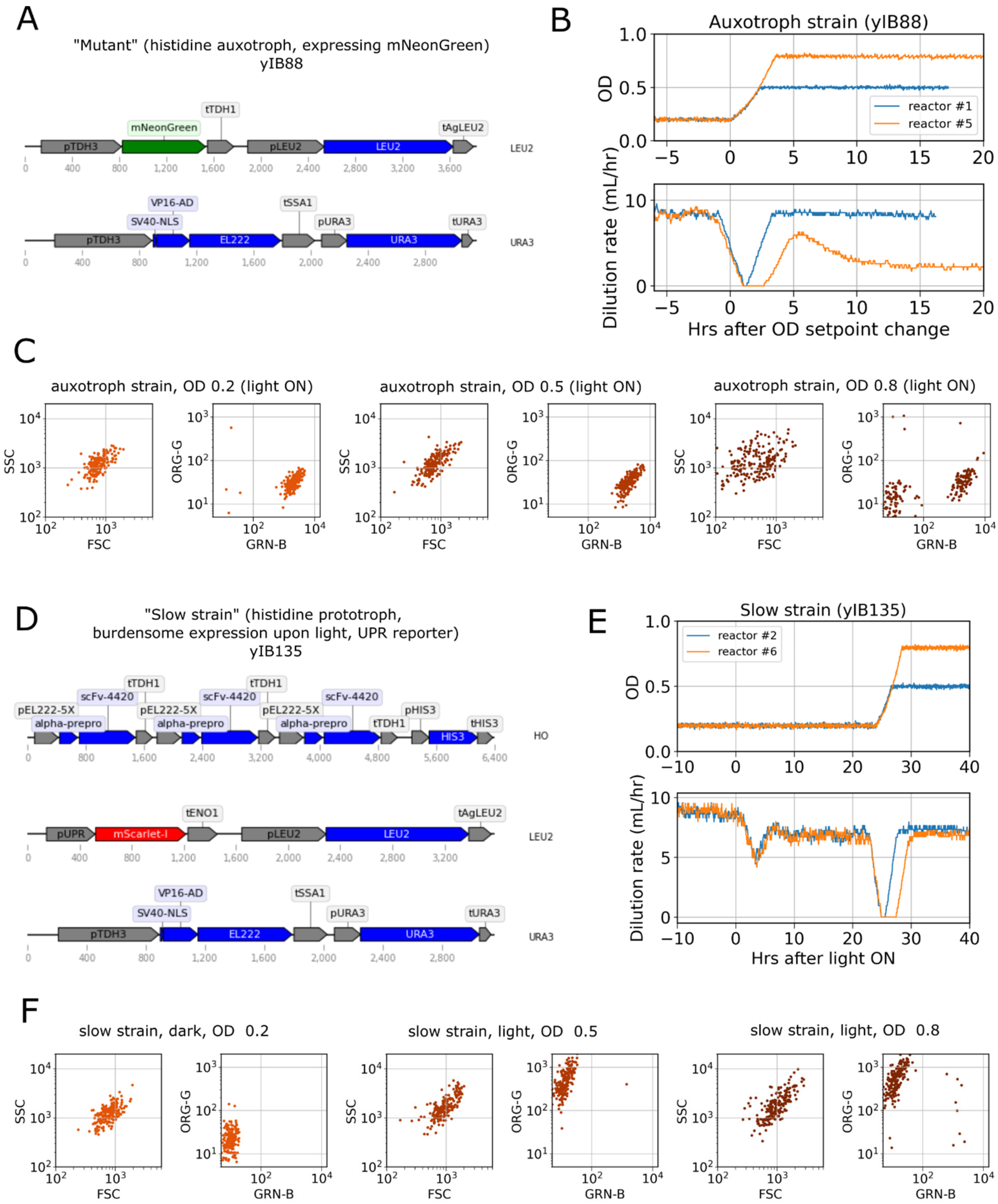

Supplementary Figure 14. Characterization of the growth, single-cell size and fluorescence phenotypes of the two consortium strains in separate turbidostat cultures. (A-C) *Genotype (A), growth rate characterization (B) and single-cell cytometry-based phenotyping (C) of the mutant strain.* ***(D-F)*** *Same as (A-C) but for the slow strain. This data correspond to four turbidostat parallel experiments with automated cytometry. It was run at the same time as 4 reactors with co-cultures*

of the two strains (Supplementary Figure 15). GRN-B and ORG-G are the best channel to respectively measure mNeonGreen and mScarlet-I fluorescence.

Strain identification strategy for the new low histidine media

A pre-requisite to attempt real-time based control of the two-strain ratio informed by cytometry data is to be able to accurately quantify this ratio from data, i.e. correctly genotyping cytometry events. We used the mono-culture controls discussed in Supplementary Figure 14 to choose an appropriate genotyping algorithm and estimate its reliability (Supplementary Figure 15).

To identify the genotype of all viable cells while ignoring un-viable cells, we chose to exclude events with too small size (FSC < 400) and also events for which the ratio between GRNB and ORG is too close to one (referred to as "unclear fluorescence", could be caused by dead or dying cells). As depicted in Supplementary Figure 15, this algorithm performs well overall. We note, however, that at high OD a small fraction of cells in auxotroph strain mono-cultures can be labelled as being the slow strain (around 7% of labelled events). A smaller fraction of labelled cells from a slow strain mono-culture exhibited high GRNB fluorescence and low ORG-G fluorescence and was thus labelled as auxotroph cells. We believe this could be due to cross-contamination at the level of cytometry acquisition caused by the high cell density used, resulting in insufficient washing of the cytometer mixer or capillary. Such issues can be solved by increasing the dilution rate when loading the cytometer plate from the culture samples and/or making the wash of the mixer and capillary by the cytometer more stringent. Due to the risk of a such fraction of misidentifications, we decided to only attempt target population ratios that are within the range of 1:3 to 3:1.

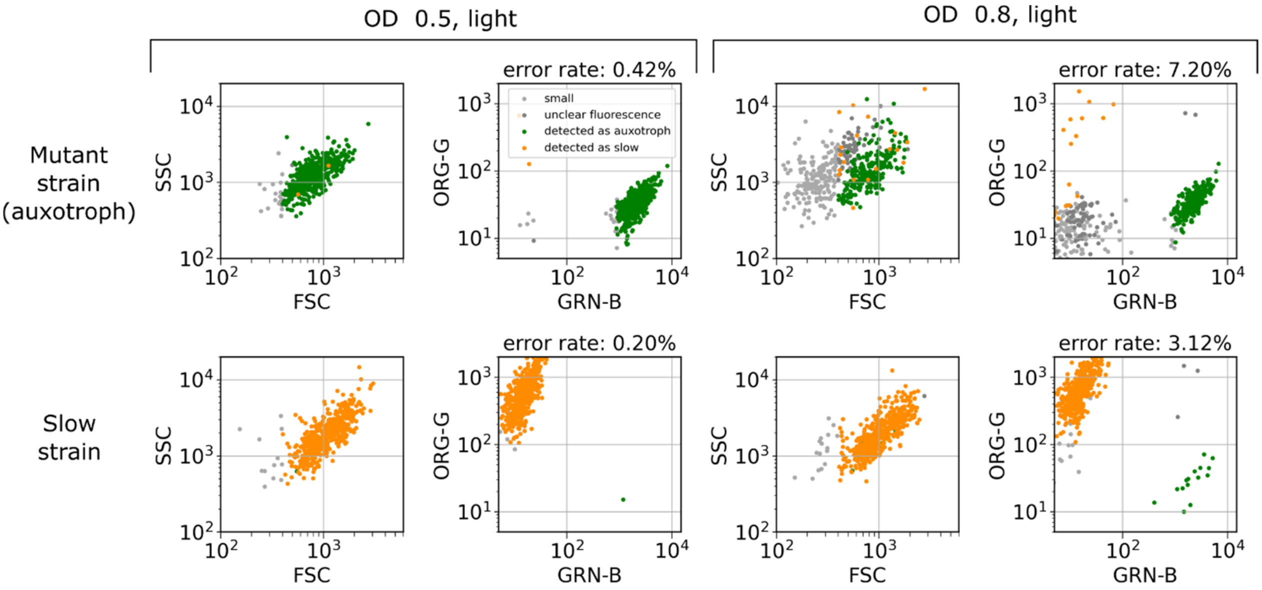

Supplementary Figure 15. Strain identification for the two-strain consortium. *Representative cytometry timepoints are shown and all events are classified based on FSC and the GRN-B/ORG-G ratio. Events with too small FSC are excluded (light gray). Events with fluorescence ratio between (0.05 and 3) are excluded (unclear fluorescence, dark gray). The rest are classified either as WT (high GRN-B/ORGG ratio) or slow (low GRN-B/ORG-G ratio).*

It should be noted that the original strain identification strategy used for the control experiment presented in Figure 4c of the main text was less sophisticated than the one presented above. Notably, it did not exclude events with small FSC; and also it counted as being cells of the slow strain genotype all events with low GRN-B or high ORG-G. Applying the improved strain identification strategy to the data of the original control experiment systematically increased the auxotroph over slow ratios,

bringing them closer to the targets. However, it did not change the fact that steering in favor of the auxotroph strain was lost after some time.

Characterization and modeling of competition dynamics in new low histidine media

We used the strain identification strategy described in Supplementary Figure 15 to analyze competition dynamics of co-cultures of the two strains with the new low histidine medium and at different OD setpoints (4 reactors, run in parallel to the 4 mono-cultures used in Supplementary Figure 14 and 15). At low OD, the auxotroph strain always wins (Supplementary Figure 16A, first time interval with a green line), as expected. When the OD setpoint is switched to higher values, an inflexion of the competition dynamics is observed, with the slow prototroph strain growing faster than the auxotroph strain at the highest ODs. We note however that there is some transient phase upon the shifts, during which the auxotroph strain is more severely slowed down. This might be due to transient dynamics of extra-cellular histidine levels in the culture, as during the time needed to reach the higher OD levels there is no fresh medium input. There could also be more complex phenomena, including change in production and consumption by the slow prototroph strain and associated cross-feeding.

We investigated the "steady-state" competition growth rate differences to relate them to the OD of the culture (Supplementary Figure 16B). In the range of OD 0.5 – 1.0, the extracted growth rate difference appeared linear. Therefore, for our control experiments we will use a model describing the growth rate difference as a linear function of the OD.

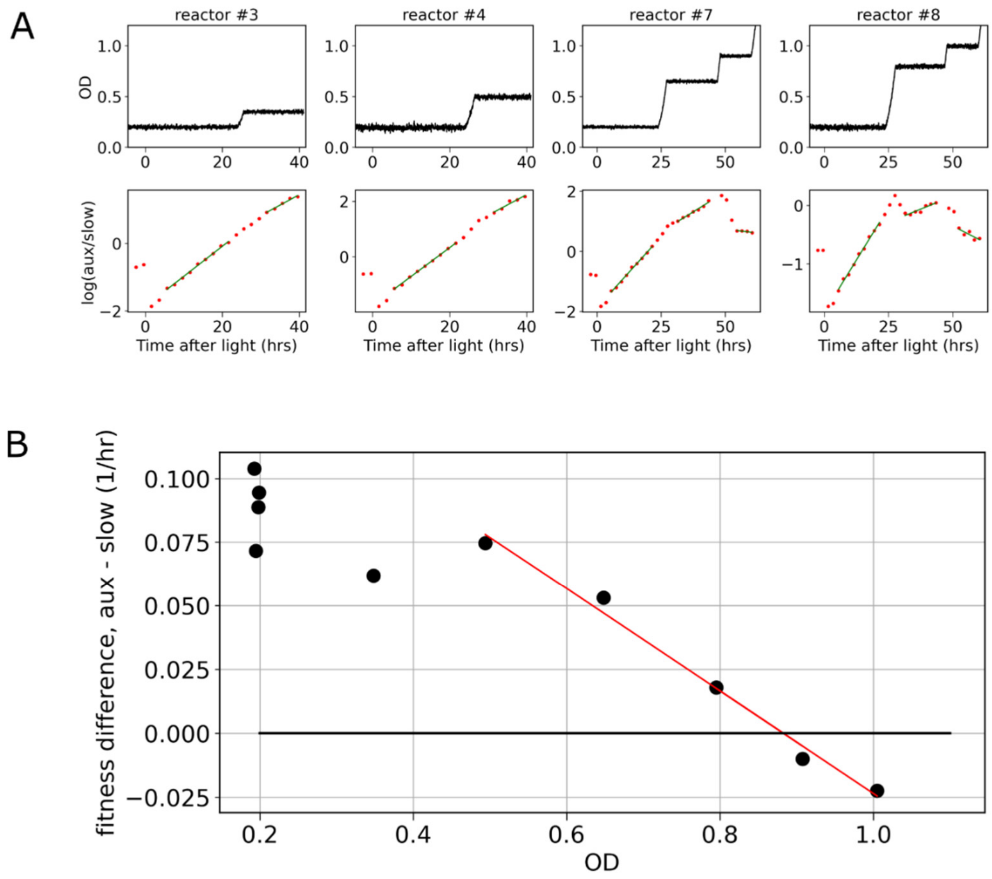

Supplementary Figure 16. Characterization and modeling of the competition dynamics. (A) Characterization data. (B) Extracted relationship between OD and the fitness (growth rate) difference between the auxotroph strain and the slow strain. A linear relationship in the OD range 0.5 to 1.0 is appearing and can be used as a model for model-predictive control.

Additional control results in new low histidine media

After the extensive characterization of the two-strain behavior in better suited medium (Supplementary Figure 14), after having improved our strain identification strategy (Supplementary Figure 15), and updated model parameterization of competition dynamics (Supplementary Figure 16), we did another attempt to control the ratio of the two strains.

We targeted three different ratios: 1:3, 1:1, and 3:1 (two reactor replicate per target). The observed ratio dynamics and the OD dynamics resulting from the controller choice are shown in Supplementary Figure 17. Clear bi-directional steering was achieved and maintained over the whole course of the experiment (> 40 hours), although the ratios oscillated around the targets. Replicate runs were similar but not identical.

Reducing the strong oscillations would require improving the model to account for delays. For example, our controller does not account for the fact that the OD cannot change immediately, especially in the upward direction which requires growth. It is also not accounting for the fact that upon a shift, a time delay is needed to reach a steady histidine concentration in the culture and then later a steady growth rate difference. Such model extensions are of interest but are beyond the scope of our current study.

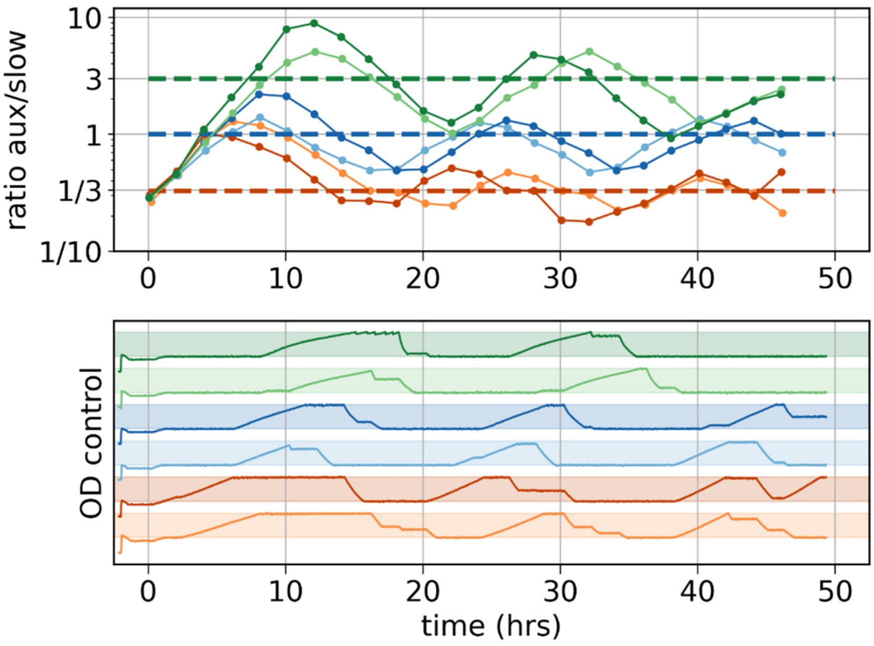

Supplementary Figure 17. Attempt to maintain the consortium composition at different ratios using dynamic control of OD using a new low histidine media. *Note that the OD range for the controller was extended ([0.5 – 1.3] instead of [0.5 – 1.0] in the characterization experiments) so as to have stronger control actions in the direction favoring the slow strain. Cytometry data were acquired every 2 hours and the strain identification strategy described in Supplementary Figure 13 was used. Just after data collection and computation of the ratio, the control algorithm optimized an OD trajectory made of 4 constant OD values corresponding to consecutive 2-hours intervals (i.e., 4-dimensional search), and the OD setpoint of the culture was changed accordingly. Note that the sample dilution made using the OT-2 robot while loading the cytometer was doubled compared to the standard dilution factor (from 20X to 40X dilution) to minimize issues with too high cell density running through the cytometer.*

Supplementary Note 4. Yeast strains and plasmids used in this study

Table 1 provides a detailed description of all yeast strains described in this study.

Name	Parent strain	URA3 locus (URA3 selection)	LEU2 locus (LEU2 selection)	HO locus (HIS3 selection)	Auxotrophies	Used in figures
yIB32	BY4741	pTDH3 NLS-VP16-EL222 tSSA1 (pIB120)			leucine, histidine, methionine	
yIB56	yIB32	pTDH3 NLS-VP16-EL222 tSSA1 (pIB120)	spacer (pIB137)		histidine, methionine	2b, 2c
yIB87	yIB32	pTDH3 NLS-VP16-EL222 tSSA1 (pIB120)	pTDH3 mCerulean tTDH1 (pIB273)		histidine, methionine	2b, 2c
yIB88	yIB32	pTDH3 NLS-VP16-EL222 tSSA1 (pIB120)	pTDH3 mNeonGreen tTDH1 (pIB274)		histidine, methionine	2b, 2c, 4c
yIB43	yIB32	pTDH3 NLS-VP16-EL222 tSSA1 (pIB120)	pTDH3 mScarlet-I tTDH1 (pIB135)		histidine, methionine	2b, 2c
yIB84	yIB32	pTDH3 NLS-VP16-EL222 tSSA1 (pIB120)	pTDH3 mCerulean tTDH1 | pTDH3 mNeonGreen tTDH1 | pTDH3 mScarlet-I tTDH1 (pIB270)		histidine, methionine	2b, 2c
yIB86	yIB32	pTDH3 NLS-VP16-EL222 tSSA1 (pIB120)	pEL222 mNeonGreen tTDH1 (pIB272)		histidine, methionine	2d, 3a, 3c
yIB330	yIB90	pTDH3 NLS-VP16-EL222 tSSA1 (pIB120)	pUPR mScarlet-I tENO1 (pIB115)	pEL222-mNeonGreen-3xFLAG-tTDH1 (pIB0682)	methionine	3d
yIB169	yIB90	pTDH3 NLS-VP16-EL222 tSSA1 (pIB120)	pUPR mScarlet-I tENO1 (pIB115)	pEL222 alpha-prepro-mNeonGreen-3xFLAG tTDH1 (pIB0399)	methionine	3d
yIB148	yIB90	pTDH3 NLS-VP16-EL222 tSSA1 (pIB120)	pUPR mScarlet-I tENO1 (pIB115)	pEL222 alpha-prepro-mNeonGreen-3xFLAG tTDH1 | pEL222 alpha-prepro-mNeonGreen-3xFLAG tTDH1 | pEL222 alpha-prepro-mNeonGreen-3xFLAG tTDH1 (pIB0369)	methionine	3d
yIB90	yIB32	pTDH3 NLS-VP16-EL222 tSSA1 (pIB120)	pUPR mScarlet-I tENO1 (pIB115)		histidine, methionine	4b
yIB119	yIB90	pTDH3 NLS-VP16-EL222 tSSA1 (pIB120)	pUPR mScarlet-I tENO1 (pIB115)	pTDH3 mCerulean tTDH1 | pTDH3 mCerulean tTDH1 (pIB267)	methionine	4b
yIB135	yIB90	pTDH3 NLS-VP16-EL222 tSSA1 (pIB120)	pUPR mScarlet-I tENO1 (pIB115)	pEL222 alpha-prepro-4420-scFv tTDH1 | pEL222 alpha-prepro-4420-scFv tTDH1 | pEL222 alpha-prepro-4420-scFv tTDH1 (pIB312)	methionine	4c

Supplementary Table 1. Genotypes of all yeast strains used in this study*. Strains are derived from BY4741 using genomic integration of integrative plasmids constructed with the Yeast Tool Kit modular cloning system (Lee et al., 2015). Names of integrative plasmids are indicated and corresponding plasmid maps (GenBank format) are available online.*
