# Supplementary material for: Enhancing bioreactor arrays for automated measurements and reactive control with ReacSight
Source: Nat Commun. 2022 Jun 11;13:3363. doi: 10.1038/s41467-022-31033-9 (PMC9188569; doi:10.1038/s41467-022-31033-9)
Supplement: Supplementary file 2 — Reporting Summary [file 41467_2022_31033_MOESM2_ESM.pdf]

nature research

Corresponding author(s): Gregory Batt
Last updated by author(s): May 18, 2022

Reporting Summary

Nature Research wishes to improve the reproducibility of the work that we publish. This form provides structure for consistency and transparency in reporting. For further information on Nature Research policies, see our Editorial Policies and the Editorial Policy Checklist.

Statistics

For all statistical analyses, confirm that the following items are present in the figure legend, table legend, main text, or Methods section.

n/a	Confirmed	
☐	☒	The exact sample size (*n*) for each experimental group/condition, given as a discrete number and unit of measurement
☒	☐	A statement on whether measurements were taken from distinct samples or whether the same sample was measured repeatedly
☒	☐	The statistical test(s) used AND whether they are one- or two-sided<br>*Only common tests should be described solely by name; describe more complex techniques in the Methods section.*
☒	☐	A description of all covariates tested
☒	☐	A description of any assumptions or corrections, such as tests of normality and adjustment for multiple comparisons
☐	☒	A full description of the statistical parameters including central tendency (e.g. means) or other basic estimates (e.g. regression coefficient) AND variation (e.g. standard deviation) or associated estimates of uncertainty (e.g. confidence intervals)
☒	☐	For null hypothesis testing, the test statistic (e.g. *F*, *t*, *r*) with confidence intervals, effect sizes, degrees of freedom and *P* value noted<br>*Give P values as exact values whenever suitable.*
☒	☐	For Bayesian analysis, information on the choice of priors and Markov chain Monte Carlo settings
☒	☐	For hierarchical and complex designs, identification of the appropriate level for tests and full reporting of outcomes
☒	☐	Estimates of effect sizes (e.g. Cohen's *d*, Pearson's *r*), indicating how they were calculated

Our web collection on statistics for biologists contains articles on many of the points above.

Software and code

Policy information about availability of computer code

Data collection	Custom software used for data collection is available in the ReacSight gitlab repository: https://gitlab.inria.fr/InBio/Public/reacsight. In addition to ReacSight (v1), we also used the OT-2 Python API (v1 and v2), GuavaSoft (v3.3), and Tecan Spark Control (v2.2).<br>We also provide in the ReacSight gitlab repository code enabling automated gating and deconvolution of cytometry data, and code enabling model-predictive control of gene expression using light.
Data analysis	Software used for data analysis is publicly available in the ReacSight gitlab repository: https://gitlab.inria.fr/InBio/Public/reacsight. We provide scripts performing data processing, analysis and plotting, enabling full reproducibility of figures generated from raw data.

For manuscripts utilizing custom algorithms or software that are central to the research but not yet described in published literature, software must be made available to editors and reviewers. We strongly encourage code deposition in a community repository (e.g. GitHub). See the Nature Research guidelines for submitting code & software for further information.

Data

Policy information about availability of data

All manuscripts must include a data availability statement. This statement should provide the following information, where applicable:

- Accession codes, unique identifiers, or web links for publicly available datasets
- A list of figures that have associated raw data
- A description of any restrictions on data availability

All the raw experimental data generated in this study have been deposited on Zenodo (https://doi.org/10.5281/zenodo.4776009). Sequences of plasmids used to construct all yeast strains are available in the ReacSight Git repository (https://gitlab.inria.fr/InBio/Public/reacsight) in the GenBank format. Source data are also provided with this paper.

Field-specific reporting

Please select the one below that is the best fit for your research. If you are not sure, read the appropriate sections before making your selection.

☒ Life sciences ☐ Behavioural & social sciences ☐ Ecological, evolutionary & environmental sciences

For a reference copy of the document with all sections, see nature.com/documents/nr-reporting-summary-flat.pdf

Life sciences study design

All studies must disclose on these points even when the disclosure is negative.

Sample size	Thanks to automation, samples were taken and analyzed every 2 hours or more frequently. Samples were analyzed using cytometry (see below for details). 5000 events were recorded so that robust estimates of the mean have been obtained after gating.
Data exclusions	No data has been excluded for analysis. For representation, a slight regularization treatment has been done with a Savitzky-Golay filter in the data of Fig 5, and a few aberrant measurements at starting times were removed (probably coming from issues with initial plate positioning) from the data of Fig 5c.
Replication	Data is highly reproducible along the duration of the experiments (see eg Fig 3d, a 5 day experiment) and across months (see eg Supplementary Fig 7, data replicated 6 months apart). Significant efforts are dedicated to metrology and replicability (see dedicated section in Supplementary Note 2).
Randomization	All experiments are fully automated so human bias in data production is absent.
Blinding	Blinding of the investigators during data collection has not been implemented since data collection is automated.

Reporting for specific materials, systems and methods

We require information from authors about some types of materials, experimental systems and methods used in many studies. Here, indicate whether each material, system or method listed is relevant to your study. If you are not sure if a list item applies to your research, read the appropriate section before selecting a response.

Materials & experimental systems

n/a	Involved in the study	
☒	☐	Antibodies
☒	☐	Eukaryotic cell lines
☒	☐	Palaeontology and archaeology
☒	☐	Animals and other organisms
☒	☐	Human research participants
☒	☐	Clinical data
☒	☐	Dual use research of concern

Methods

n/a	Involved in the study	
☒	☐	ChIP-seq
☐	☒	Flow cytometry
☒	☐	MRI-based neuroimaging

Flow Cytometry

Plots

Confirm that:

☒ The axis labels state the marker and fluorochrome used (e.g. CD4-FITC).

☒ The axis scales are clearly visible. Include numbers along axes only for bottom left plot of group (a 'group' is an analysis of identical markers).

☐ All plots are contour plots with outliers or pseudocolor plots.

☒ A numerical value for number of cells or percentage (with statistics) is provided.

Methodology

Sample preparation	Yeast cells were acquired directly from bioreactor cultures after dilution using DI water with the OT-2 robot. We convert raw cytometry data into fluorophore concentrations in relative promoter units using a pipeline described in Supplementary Note 2 and in Supplementary Figure 7. It uses data from single color strains with pTDH3-driven expression for normalization.
Instrument	Guava EasyCyte 14 HT BGV

Software	Custom code was used for gating and spectral deconvolution and is available in the ReacSight repository https://gitlab.inria.fr/InBio/Public/reacsight.
Cell population abundance	We work with yeast cultures. No abundance issues were encountered.
Gating strategy	To enable reactive experiment control based on cytometry data, we developed and implemented algorithms to perform automated gating and spectral deconvolution between overlapping fluorophores. Data from several channels are used to provide an improved estimate of the level of fluorescent proteins. This is shown in Figure 2b and described in details in Supplementary Note 2 and Supplementary Figure 7.

☒ Tick this box to confirm that a figure exemplifying the gating strategy is provided in the Supplementary Information.
