# Supplementary material for: Enhancing bioreactor arrays for automated measurements and reactive control with ReacSight
Source: Nat Commun. 2022 Jun 11;13:3363. doi: 10.1038/s41467-022-31033-9 (PMC9188569; doi:10.1038/s41467-022-31033-9)
Supplement: Supplementary file 5 — Description of Additional Supplementary Files [file 41467_2022_31033_MOESM5_ESM.pdf]

Title: Supplementary Movie 1

Description: The movie illustrates how to use ReacSight to set up experimental platforms and highlights key steps of the functioning of a bioreactor-based platform.
